# Rapid Screening for Flavone *C*-Glycosides in the Leaves of Different Species of Bamboo and Simultaneous Quantitation of Four Marker Compounds by HPLC-UV/DAD

**DOI:** 10.1155/2012/205101

**Published:** 2012-05-10

**Authors:** Jin Wang, Yong-de Yue, Hao Jiang, Feng Tang

**Affiliations:** SFA Key Laboratory of Bamboo and Rattan Science and Technology, International Centre for Bamboo and Rattan, No. 8 Futong Dongdajie, Wangjing, Chaoyang District, Beijing 100102, China

## Abstract

A strategy for analyzing flavone *C-*glucosides in the leaves of different species of bamboo was developed. Firstly, the flavone *C-*glycosides were extracted from the bamboo leaves (51 species in 17 genera) with methanol and chromatographed on silica gel 60 plates in automatic developing chamber (ADC2), and a qualitative survey using simple derivatization steps with the NP reagent was carried out. The flavone *C-*glycosides were found in 40 of 51 species of bamboo examined. Secondly, an HPLC method with photodiode array and multiple wavelength detector was optimized and validated for the simultaneous determination of flavone *C-*glycosides, including isoorientin, isovitexin, orientin, and vitexin in the leaves of three species of bamboo and the flavone *C-*glycosides were confirmed by LC/MS. The optimized HPLC method proved to be linear in the concentration range tested (0.2–100 *μ*g/mL, *r* ^2^ ≥ 0.9997), precise (RSD ≤ 1.56%), and accurate (88–106%). The concentration ranges of isoorientin, isovitexin, orientin, and vitexin in three bamboo leaves samples were 1.00–2.78, 0–0.31, 0–0.07, and 0.20–0.68 mg/g, respectively. The proposed method was validated to be simple and reliable and can be a tool for quality control of bamboo leaf extract or its commercial products.

## 1. Introduction

**Figure 1:**
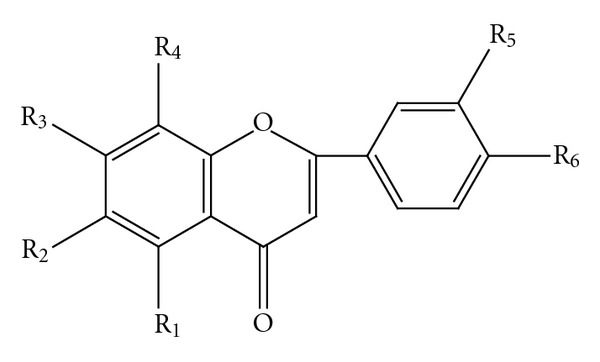
Chemical structures of isoorientin, isovitexin, orientin, and vitexin. Isoorientin: R_1_ = R_3_ = R_5_ = R_6_ = OH, R_4_ = H, R_2_ = glucose; isovitexin: R_1_ = R_3_ = R_6_ = OH, R_4_ = R_5_ = H, R_2_ = glucose; orientin: R_1_ = R_3_ = R_5_ = R_6_ = OH, R_2_ = H, R_4_ = glucose; vitexin: R_1_ = R_3_ = R_6_ = OH, R_2_ = R_5_ = H, R_4_ = glucose.

Bamboo leaves have been used in traditional Chinese medicine for treating fever and detoxification for over 1000 years. It was found that extract of bamboo leaves has multiple biological activities, such as cancer prevention [1, 2], anti-free radical, and antioxidation [3, 4], and can be used as a pharmaceutical intermediate and food additive. A new product named bamboo-leaf flavonoid has appeared on the market. The main functional components of the extract from bamboo leaf are flavone *C*-glycosides including isoorientin, isovitexin, orientin, and vitexin (Figure 1), which have been used as marker compounds for the determination of commercial products of bamboo-leaf flavonoids.

Bamboo is an important renewable resource and more than 1250 bamboo species, belonging to 75 genera, are distributed all over the world [5]. The quality of different batches of the same herb could vary significantly [6]. Hence, to investigate and determine these flavone *C*-glycosides in the leaves of different species of bamboo is needed.

Thin-layer chromatography (TLC) chemical screening is simple, fast, and of low cost in identification of chemical ingredient from plant extracts [7, 8]. Many papers about TLC chemical screening have been published in recent years [9, 10]. Analytical methods for determination of the *C-*glycosylflavone isomer pairs orientin/isoorientin and vitexin/isovitexin, including HPLC [11], HPCE [12], HPTLC [13, 14], and HPLC-MS [15], have been reported. However, the reported HPLC chromatographic conditions need to be optimized to obtain a good resolution of adjacent peaks. Furthermore, HPLC analysis for selected bamboo species in this study was not reported.

The work aimed to develop a two-step procedure to quickly survey a large number of bamboo leave samples (51 species in 17 genera) for the presence of flavone *C-*glycosides and to optimize and validate an HPLC method for the simultaneous quantitation of marker compounds in different bamboo leaves and its commercial products.

## 2. Experimental

### 2.1. Plant Material and Reagents

The leaves of fifty-one bamboo species were collected from five regions in China in 2007 and authenticated by Professor Jiusheng Peng from Jiangxi Academy of Forestry, Professor Yulong Ding from Nanjing Forestry University, and Professor Qirong Guo from International Centre for Bamboo and Rattan, respectively. Their family, species, and plant sources are shown in Table 1. Bamboo leaves were dried in the shade, ground to powder, and stored below −20°C.

The HPLC-grade acetonitrile and methanol were purchased from fisher scientific (USA). Glacial acetic acid (≥99.7%) was purchased from Sigma-Aldrich (USA). Water was purified with an ultrapure water system (Purelab Plus, Pall, USA). Analytical-grade chemical was obtained from Beijing Chemical Works (China). Commercial products of bamboo-leaf flavonoids labeled with total content of flavonoids (30%) and antioxidant potency were obtained from Jinhua Beikang Biochemical Development Co., Ltd. (Zhejiang, China). The standard compounds of isoorientin, orientin, isovitexin and vitexin were purchased from Shanghai Winherb Medical S & T Development Co., Ltd. (Shanghai, China).

### 2.2. Preparation of Standard Solutions

Isoorientin and isovitexin stock solutions were prepared in methanol at the concentration of 370 *μ*g/mL and 480 *μ*g/mL. Orientin and vitexin stock solutions were prepared in methanol/water (90/10, v/v) at the concentration of 480 *μ*g/mL and 440 *μ*g/mL. The standard solutions were prepared by transferring appropriate volumes of stock solutions of each compound to a 10 mL volumetric flask and diluting to volume with methanol/water (60/40, v/v). A series of standard solutions with concentrations 0.01–100 *μ*g/mL were prepared.

### 2.3. TLC Screening of Flavone *C*-Glycosides

The dried bamboo leaf powder (1.0 g) was extracted by percolation with 20 mL of methanol and then sonicated for 30 min at room temperature. Thin-layer chromatography was performed on the plates precoated with silica gel 60 F254 (Merck, Germany). Samples (4 *μ*L) were applied to the plate as bands of 8 mm wide by means of a Camag (Switzerland) Linomat V sample applicator. The plate was developed in an automatic developing chamber (ADC2) (Camag, Switzerland) using ethyl acetate : formic acid : water (82 : 9 : 9, v/v/v) as the mobile phase. The developing distance was 80 mm. After development, plates were dried and derivatisation was carried out by immersion of the plates in a solution of NP reagent (diphenylborinic acid 2-aminoethyl ester (1.0 g) in ethyl acetate (200 mL) and PEG 400 (20 g) in dichloromethane (400 mL) [16].

### 2.4. Sample Preparation for HPLC and LC/MS Analysis

Dried bamboo leaves powder (1.0 g) was accurately weighed and transferred in a 150 mL conical flask with glass stopper, and 60% aqueous methanol (15 mL) was added [3, 17]. The solution was stirred for 12 h and sonicated for 30 min at room temperature and then centrifuged at 5000 rpm for 5 min. The residue was further extracted with 60% methanol (2 × 15 mL) as mentioned above. All the extracted solutions were combined in a 50 mL volumetric flask and diluted to the mark. All solutions were filtered through 0.45 *μ*m membrane filter prior to analysis. An aliquot of 20 *μ*L of solution was injected for HPLC and LC/MS analysis.

### 2.5. HPLC Analysis

The HPLC-UV/DAD analyses were carried out on a Waters Alliance 2695 (Milford, MA, USA) system connected to a model 2996 (DAD). The separation was performed using a C_18_ column (4.6 × 250 mm, 5 *μ*m, YMC pack R&D ODS-A, YMC Japan) at 30°C. The solvent system consisted of a mixture of water with 0.5% (v/v) glacial acetic acid and acetonitrile (85/15, v/v) at a flow rate of 1.0 mL/min.

### 2.6. LC/MS Analysis

The LC-MS analysis was conducted using an Agilent 1100 system equipped with a photodiode array and multiple wavelength detector. The column and the chromatographic condition were the same as those used for the HPLC analysis. The LC effluent was split using a T-splitter to produce a flow of 0.25 mL/min. The identification of isoorientin, isovitexin, orientin, and vitexin was performed on the quadrupole mass spectrometer equipped with electrospray ionization (ESI-MS) in the negative mode. The collision energy was 60 eV. Capillary voltage was 3.5 kV, and the drying gas was set at 350°C. The scan range was set at *m/z *100 to 1000.

### 2.7. Validation of HPLC Method

The HPLC method was validated in terms of linearity, limit of detection (LOD), limit of quantitation (LOQ) precision, specificity, and accuracy according to the International Conference on Harmonization (ICH) guidelines [18].

The calibration curves were constructed by plotting peak area versus concentrations with the help of the Empower software. The standard solutions of the analytes were diluted with 60% methanol to yield a series of appropriate concentrations. LOD and LOQ were estimated experimentally by injecting a series of dilute solutions with known concentrations until the signal-to-noise ratio for the standards reached a 3 : 1 ratio for LOD and 10 : 1 for LOQ.

Intraday and interday variations were chosen to determine the precision of the HPLC method. For intraday precision, three different concentrations of standard solutions (5, 15, and 50 *μ*g/mL) were determined in one day and expressed as relative standard deviation (RSD). For interday precision, the standards were determined for three consecutive days.

The specificity of the method was ascertained by analyzing standard compounds and samples. The peaks for the marker compounds from sample solutions were confirmed by comparing the retention time and UV spectra with the reference standards. The peak purity was checked using DAD (*λ* = 210–400 nm). The peaks were considered pure when there was a coincidence between the two spectral sections.

Recovery experiments were performed to evaluate the accuracy of the method. The extract of bamboo leaves used for recovery studies was preanalyzed and spiked with the mixed standards at high, middle, and low concentration levels. Three replicates were performed for the test and variations were expressed by standard deviation (SD).

## 3. Results and Discussion

### 3.1. Result of Preliminary TLC Screening

**Figure 2:**
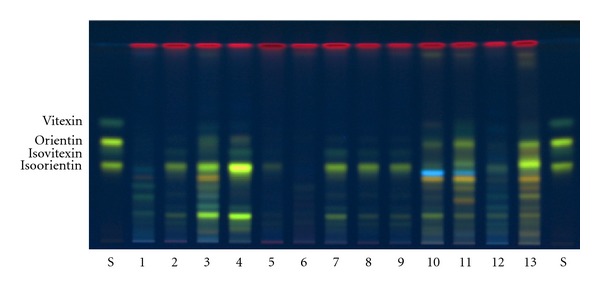
TLC profile of standards and extracts from the different species of bamboo. S: standard mixture: isoorientin (*R*_*f*_ = 0.39), isovitexin (*R*_*f*_ = 0.47), orientin (*R*_*f*_ = 0.51), and vitexin (*R*_*f*_ = 0.61). Different bamboo species are listed in Table 1.

To optimize the TLC chromatographic conditions, different mobile phases and derivatisation regents were evaluated. The mobile phase ethyl acetate : formicacid : water (82 : 9 : 9, v/v/v) gave a good seperation of the markers. Satisfactory derivatisation efficiency was obtained using diphenylborinic acid 2-aminoethyl ester and PEG 400 as derivatisation regents. After derivatisation, flavone *C-*glycosides including isoorientin, isovitexin, orientin, and vitexin showed the four characteristic fluorescent bands on the TLC plates with the colour of yellow, green, yellow and green, respectively. A typical TLC profile of extracts from bamboo leaves is shown in Figure 2.

The flavone *C-*glycosides, especially isoorientin, were found in most of these species of bamboo tested. The different species of bamboo showed different major constituents. Identification of isoorientin, isovitexin, orientin, and vitexin in the different species of bamboo was carried out by the same retention factors (*R*_*f*_) and similar colour to the standards compounds. The results of the flavone *C*-glycosides survey are presented in Table 1. The flavone *C-*glycosides were found in 40 of 51 species of bamboo examined. TLC screening method proved to be an efficient strategy for the rapid screening of flavone *C-*glucosides. Among the fifty-one species of bamboo, *B. multiplex *cv. *Silverstripe*, *P. heterocycla *cv. *pubescens,* and *P. acuta* showed strong band intensity as to flavone *C-*glycosides on the TLC plates, so they were examined further by HPLC.

### 3.2. Optimization of HPLC Conditions

**Figure 3:**
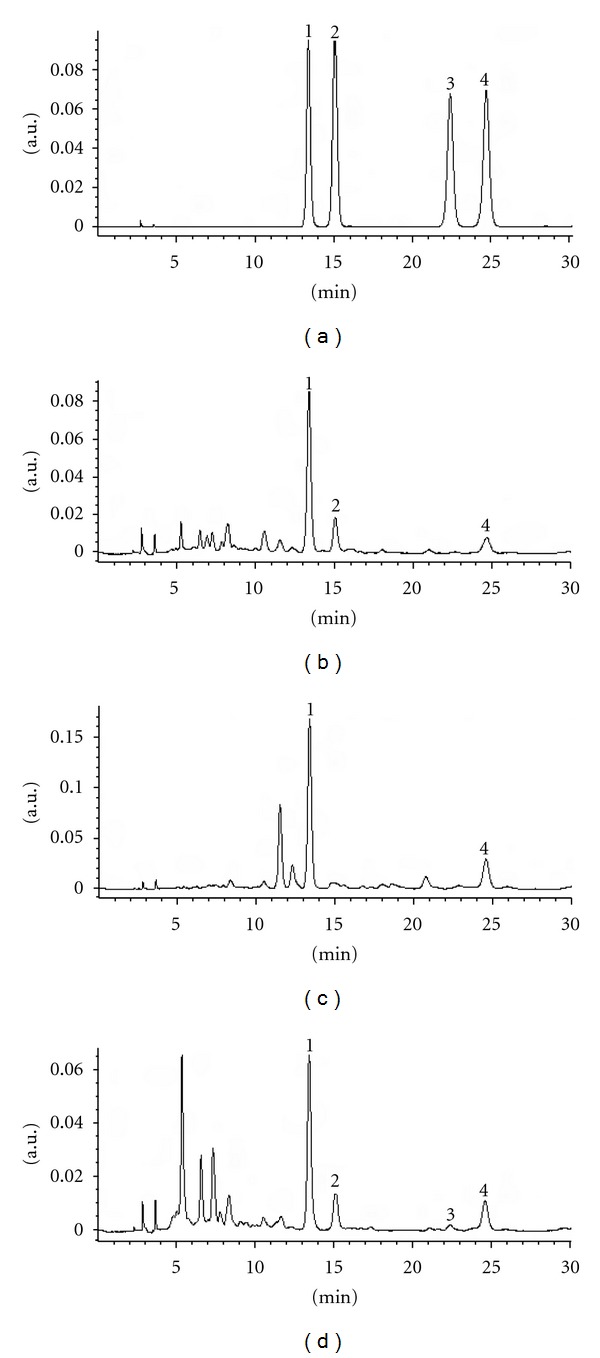
HPLC chromatograms of the standard mixture (a), 60% methanol extract of *P. acuta* (b), *B. multiplex* cv.* Silverstripe *(c), and *P. heterocycla *cv. *pubescens* (d) using diode array detection at 345 nm. (1) Isoorientin, (2) orientin, (3) vitexin, and (4) isovitexin.

To achieve a good chromatographic resolution, different types of chromatographic columns such as Xterra RP18 (4.6 × 250 mm, 5 *μ*m; Waters USA), Zorbax SB-C18 (4.6 × 250 mm, 5 *μ*m; Agilent USA), and YMC-Pack R&D ODS-A (4.6 × 250 mm, 5 *μ*m; YMC Japan) were tested. To enhance the resolution and eliminate the peak tailing of the marker compounds, glacial acetic acid was added to the mobile phase. Different mobile phase compositions and different column temperatures were also optimized. The best results were obtained using a YMC-Pack R&D ODS-A column at 30°C, with acetonitrile and water containing 0.5% (v/v) glacial acetic acid as the mobile phase and isocratic elution (1.0 mL/min) was applied (Figure 3(a)).

### 3.3. Validation of HPLC Quantitation Method

Standard solutions of isoorientin, orientin, vitexin, and isovitexin were prepared at a concentration range of 0.2–100 *μ*g/mL. The nine-point calibration curves of the analytes showed good linearity (*r* ^2^ ≥ 0.9997) with given concentration ranges. The parameters of linearity and range were summarized in Table 2, and LOD and LOQ values showed the adequate sensitivity of the proposed method. 

The RSD values for both parameters ranged from 0.05 to 1.56% (Table 3). Therefore, the method was considered repeatable. 

 The recovery values of the four flavone *C*-glycosides are given in Table 4. Average recoveries ranged from 88.44% to 105.83%, confirming the accuracy of the method. 

**Figure 4:**
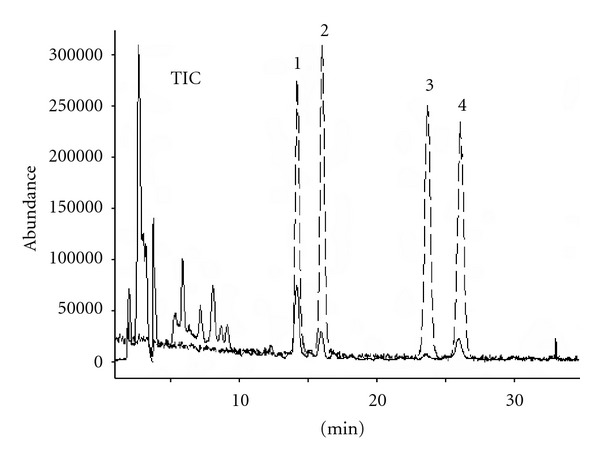
Overlap of TIC profiles (LC/MS ESI, negative mode) of mixed standards (dashed line), and the 60% methanol extract of *Phyllostachys heterocycla* cv. *pubescens *(solid line). For the identification of 1–4, see Table 5.

 The four flavone *C*-glycosides in bamboo leaves were identified by comparing their UV/DAD and MS spectra with those of the standards (Figures 3 and 4). The result of peak purity test showed that there was no coelution to interfere with the quantitation of marker compounds. 

The four flavone *C-*glycosides from bamboo leaves were detected. The retention time, maximum wavelength, and characteristic fragment ions for individual peaks are presented in Table 5. 

### 3.4. Sample Analysis

The optimized method was applied to determinate the four marker compounds in different samples. These HPLC chromatograms are depicted in Figure 3. The results are shown in Table 6. The concentration ranges of isoorientin, isovitexin, orientin, and vitexin in three bamboo leaves samples were 1.00–2.78, 0–0.31, 0–0.07, and 0.20–0.68 mg/g, respectively. A total of four flavone *C-*glycosides were found in the leaf of *P. heterocycla *cv. *Pubesceus*, which has been used as a raw material to produce commercial products of bamboo-leaf flavonoids. The quantitative data also showed that the content of isoorientin in *B. multiplex *cv. *Silverstripe* was higher than that in the two other bamboo species. It could be considered a promising plant source for preparation of isoorientin.

## 4. Conclusion

In summary, this paper describes a two-step procedure for the rapid screening of flavone *C*-glucosides in a large number of samples and quantitative analysis of four marker compounds. The proposed procedure proved to be specific and sensitive and can be regarded as an alternative method to detect and quantify flavone *C*-glucosides in bamboo leaves and its commercial product.

## Figures and Tables

**Table 1:** Results of TLC screening for isoorientin, isovitexin, orientin, and vitexin in the leaves of 51 different species of bamboo.

No.	Family	Bamboo species	Bands intensity^a^				Plant sources^b^
			Isoorientin	Isovitexin	Orientin	Vitexin	
1	*Acidosasa*	*A. gigantea*	−	−	−	−	A
2	*Bambusa*	*B. multiplex *cv.* Alphonse-Karr *	+	+	−	−	B
3		*B. textilis *McClure	+	−	+	−	A
4		*B. multiplex *cv. *Silverstripe *	+	+	−	−	A
5		*B. ventricosa *McClure	+	−	−	−	A
6		*B. vulgaris *cv.* Wamin *	−	−	−	−	A
7		*B. multiplex *var.* multiplex *	+	+	−	−	B
8		*B. multiplex *cv. *Fernleaf *	+	+	−	−	A
9		*B. multiplex *var.* riviereorum* R. Maire	+	+	−	−	A

10	*Bashania*	*B. fargesii* (E. G. Camus) Keng f. et Yi	+	−	+	+	B

11	*Brachystachyum*	*B. densiflorum* (Rendle) Keng	+	−	+	+	A

12	*Chimonobambusa*	*Ch. quadrangularis *(Fenzi) Makino	+	−	−	−	A

13	*Chimonocalamus*	*Chi. Delicates*	+	−	+	−	C

14	*Dendrocalamus*	*D. oldhamic *McClure	+	−	−	−	A
15		*D. latiflorus *Munro	−	−	−	−	D
16		*D. minor *var*. amoenus *(Q. H. Dai et C. F. Huang) Hsueh et D. Z. Li	+	+	−	+	E
17		*D. hamiltonii* Nees et Arn. ex Munro	−	−	−	−	D
18		*D. yunnanicus* Hsuch et	−	−	−	−	E

19	**Indocalamus**	**I. Tessellatus **(Munro) Keng f.	+	−	−	−	E
20		**I. *latifolius* (Keng) McClure	+	−	−	−	E
21		*I. longiauritus* Hand.-Mazz	−	−	−	−	E

22	*Indosasa*	*I. sinica* C. D. Chu et C. S. Chao	−	−	−	−	A

23	*Menstruocalamus*	*M. sichuanensis*	+	−	+	−	B

24	*Neosinocalamus*	*N. affinis* (Rendle) Keng f.	+	−	−	−	E

25	*Phyllostachys*	*P. meyeri* McClure	−	−	−	−	E
26		*P. nidularia *Munro	+	−	+	−	A
27		*P. aureosulcata* cv.* spectabilis *	+	−	+	−	A
28		*P. glauca *McClure	+	−	+	−	B
29		*P. dulcis* McClure	+	−	−	−	A
30		*P. acuta* C. D. Chu et C. S. Chao	+	+	+	−	A
31		*P. *stimulosa ** H. R. Zhao et A. T. Liu	−	−	+	−	A
32		*P. heteroclada *Oliver	−	−	−	−	A
33		*P. sulphurea* cv.* Houzeau *	+	+	−	−	A
34		*P. heterocycla *cv. *pubescens *	+	+	+	+	A
35		*P. vivax *McClure	+	−	+	−	A
36		*P. mannii* Gamble	+	−	+	−	A
37		*P. bambusoides* f.* shouzhu *	+	−	+	−	A
38		*P. aurea* Carr.ex A. et C. Riviere	+	−	+	−	A

39	*Pleioblastus*	*P. maculatus *(McClure) C. D. Chu et C. S. Chao	+	−	+	−	B
40		*P. amarus *(Keng) Keng f.	−	−	+	+	B
41		*P. gramineus *(Bcan) Nakai	+	−	+	−	B
42		*P. gozadakensis* Nakai	+	−	+	−	B
43		*P. yixingensis*	−	−	—	−	B

44	*Pseudosasa*	*P. cantori *(Munro) Keng f.	−	−	—	−	A
45		*P. japonica *(Sieb. et Zucc.) Makino	−	−	+	−	B
46		*P. amabilis *(McClure) Keng f.	+	−	+	−	A

47	*Sasa*	*S. fortunei *(Van Houtte) Fiori	+	−	+	−	B
48		*S. pygmaea* (Miq.) E. G. Camus	+	−	+	−	B
49		*S. argenteastriatus *E. G. Camus	+	−	+	−	B

50	*Shibataea*	*Sh. chinensis* Nakai	+	−	+	−	B

51	*Sinobambusa*	*Si. tootsik* (Sieb.) Makino	−	−	+	−	B

^a^+ Presence. − not detected or absence;

^b^A: Bamboo Garden of Jiangxi Academy of Forestry (Nanchang, China);

B: Bamboo Garden of Nanjing Forestry University (Nanjing, China);

C: Forestry Bureau of Jinping county (Yunnan, China);

D: Xishuangbanna Tropical Bamboo Garden (Yunnan, China);

E: Changning Century Bamboo Garden (Sichuan, China).

**Table 2:** Regression equations, linear range, LOD, and LOQ for quantitative analysis by HPLC (*n* = 3).

Compound	Linear range (*μ*g/mL)	Regression equation^a^	Correlation coefficient (*r* ^2^)	LOD (*μ*g/mL)	LOQ (*μ*g/mL)
Isoorientin	0.2–100	*Y* = 2240*X* − 4620	0.9997	0.02	0.07
Orientin	0.2–100	*Y* = 2590*X* − 6080	0.9997	0.02	0.07
Vitexin	0.2–100	*Y* = 2600*X* + 516	0.9998	0.03	0.10
Isovitexin	0.2–100	*Y* = 2830*X* + 3430	0.9998	0.02	0.07

^a^ *Y* is the peak area; *X* is the amount (ng).

**Table 3:** Intra- and interday precision by the HPLC quantitation method.

Compound	Concentration (*μ*g/mL)	Intraday (*n* = 4) RSD (%)			Interday (*n* = 12) RSD (%)
		Day 1	Day 2	Day 3	
Isoorientin	5	0.77	0.66	0.74	1.12
	15	0.62	0.08	0.15	0.90
	50	0.13	0.08	0.14	0.73

Orientin	5	0.64	0.27	0.18	1.18
	15	0.30	0.08	0.17	0.77
	50	0.21	0.08	0.08	0.73

Vitexin	5	0.16	0.15	0.22	0.64
	15	0.09	0.07	0.05	0.57
	50	0.25	0.06	0.08	0.57

Isovitexin	5	0.29	0.20	0.27	0.66
	15	1.56	0.11	0.16	1.33
	50	0.31	0.07	0.12	0.64

**Table 4:** Recoveries of isoorientin, isovitexin, orientin, and vitexin by the HPLC method.

Bamboo species	Compound	Original contents (*μ*g/mL)	Spiked (*μ*g/mL)	Amount found (*μ*g/mL)	Recovery^a^ (%)	RSD (%)
*Bambusa multiplex * cv. *Silverstripe *	Isoorientin	39.79	21.26	59.38	92.16	2.33
		39.79	42.52	81.80	98.80	1.11
		39.79	59.53	96.74	95.76	2.18
	Isovitexin	9.46	4.00	13.16	92.42	3.25
		9.46	8.00	17.45	99.92	0.81
		9.46	11.20	20.54	98.96	4.66

*Phyllostachys heterocycla * cv. *Pubesceus *	Isoorientin	15.18	7.59	2.81	100.53	3.02
		15.18	15.18	29.72	95.76	5.01
		15.18	22.77	37.78	99.25	1.53
	Orientin	3.89	2.00	6.01	105.83	3.22
		3.89	4.00	7.90	100.17	5.25
		3.89	6.00	10.23	105.61	4.03
	Vitexin	0.96	0.50	1.41	89.33	7.20
		0.96	1.00	1.93	96.67	7.48
		0.96	1.50	2.29	88.44	4.90
	Isovitexin	4.02	2.00	6.12	105.00	4.23
		4.02	4.00	8.20	104.42	2.88
		4.02	6.00	10.24	103.61	4.00

*Phyllostachys acuta*	Isoorientin	20.77	11.00	31.07	93.61	1.10
		20.77	22.00	41.72	95.21	3.92
		20.77	33.00	52.71	96.79	3.98
	Orientin	4.30	2.00	6.33	101.50	5.55
		4.30	4.00	7.89	89.75	4.69
		4.30	6.00	10.11	96.83	3.48
	Isovitexin	2.84	2.50	5.07	89.07	6.13
		2.84	5.00	7.89	101.00	4.98
		2.84	7.50	9.73	91.87	3.53

^a^mean ± SD (*n* = 3).

**Table 5:** Identification of flavone *C-*glycosides from bamboo leaves.

No.	RT (min)	[M-H]^−^ (*m/z*)	[2M-H]^−^ (*m/z*)	UV *λ*_max_ (nm)	Identification
1	14.09	447.1	895.2	270, 349	Isoorientin
2	15.89	447.1	895.2	256, 349	Orientin
3	23.55	431.0	863.2	269, 338	Vitexin
4	25.93	431.0	863.2	270, 338	Isovitexin

**Table 6:** Content of isoorientin, isovitexin, orientin, and vitexin in different samples by HPLC method.

Compound	Content of marker compounds^a^ (% w/w)			
	*Bambusa multiplex * cv. *Silverstripe *	*Phyllostachys heterocycla * cv. *pubescens *	*Phyllostachys acuta * C. D. Chu et C. S. Chao	Commercial product of bamboo-leaf flavonoids
Isoorientin	0.278 ± 0.0021	0.100 ± 0.0037	0.138 ± 0.0096	2.235 ± 0.0100
Orientin	ND	0.026 ± 0.0032	0.031 ± 0.0008	0.762 ± 0.0076
Vitexin	ND	0.007 ± 0.0004	ND	0.183 ± 0.0014
Isovitexin	0.068 ± 0.0006	0.028 ± 0.0012	0.020 ± 0.0011	0.547 ± 0.0029

^a^mean ± SD (*n* = 3).
